# Protective effect of cannabinoids on gastric mucosal lesions induced by water immersion restrain stress in rats

**DOI:** 10.22038/ijbms.2021.54338.12213

**Published:** 2021-09

**Authors:** Rania Elgohary, Rania M. Abdelsalam, Omar ME Abdel-Salam, Mahmoud M. Khattab, Neveen A. Salem, Zakaria A. El-Khyat, Fatma A. Morsy

**Affiliations:** 1 Narcotics, Ergogenics and Poisons Department, National Research Centre (NRC), Cairo, Egypt; 2 Pharmacology and Toxicology Department, Faculty of Pharmacy, Cairo University, Cairo, Egypt; 3 Medical Biochemistry Department, National Research Centre (NRC), Cairo, Egypt; 4 Clinical Pathology Department, National Research Centre (NRC), Cairo, Egypt

**Keywords:** Anti-oxidant, Cannabinoid receptor, Stress, TNF-α, Ulcer

## Abstract

**Objective(s):**

This study aimed to determine the impact of cannabinoid agonists and antagonists on the mucosal lesion progress in the stomach induced by water-immersion restraint stress (WIRS).

**Materials and Methods:**

Rats subjected to WIRS for 4 hr were treated with Dimethyl sulfoxide (DMSO), CBR1 agonist (NADA, 1 mg/kg), CBR1 antagonist (Rimonabant, 1 mg/kg), CBR2 agonist (GW405833 1 mg/kg) or CBR2 antagonist (AM630, 1 mg/kg SC) 30 min before WIRS. Microscopic lesions, oxidative stress, inflammatory cytokines biomarkers, and (Myeloperoxidase) MPO in gastric tissues were determined.

**Results:**

Results indicated development of severe gastric lesions with a substantial increase in the contents of (nitric oxide) NO, (malondialdehyde) MDA, (interleukin-1 beta) IL-1β, MPO, (tumor necrosis factor-alpha) TNF-α, and a significant fall in the content of GSH and the activity of PON-1 after WIRS.

**Conclusion:**

Treatment with NADA and AM630 protected gastric tissues against ulcers as demonstrated by a decrease in the contents of MDA, TNF-α, MPO, and IL-1β along with an increase in the content of PON-1 activity and GSH in the stomach tissues. On the other hand, treatment with SR141716A or GW405833 showed no protective effects on ulcers development. It seems that cannabinoids exert their antioxidant potential and anti-inflammatory effects against WIRS-induced gastric ulcers by activation of CB1R.

## Introduction

Gastric ulcers, considered one of the most widespread gastrointestinal tract disorders, are being defined by an imbalance between pepsin and acid and insufficient mucosal response resulting in the formation of stomach lesions (1). Risk factors for developing peptic ulcers include reactive oxygen species (ROS), *Helicobacter pylori* infection, proinflammatory cytokines, Non-Steroidal Anti-Inflammatory Drugs (NSAIDs), stress, and smoking (2). Another group of defense mechanisms such as gastric mucus, bicarbonate secretion, and prostaglandins, prevents the back-diffusion of H+ derived from the lumen, and maintaining adequate blood flow plays a crucial role in preserving the stomach mucosa’s integrity (3).

Stress ulceration causes diffused lesions through the stomach’s mucosal layer, which arises from the main stressful events for example burns, shock, and severe trauma (4). Among different animal models of stress, water immersion and restraint stress, which consists of both physical and psychological stress, are commonly used as a model for stress ulceration (5). Under water-restraint stress (WRS) conditions, production of ROS results in oxidative stress which is responsible for the development of gastric mucosal ulceration (6). The stress model is widely utilized to investigate the mechanisms of stress-induced stomach ulcers in humans. The levels of pro-inflammatory cytokines have been shown to be elevated in gastric mucosa of rats that are exposed to water-immersion restraint stress (WIRS) (7). Furthermore, neutrophil infiltration in gastric tissues plays a key role in the development of stomach ulcers which is checked by Myeloperoxidase (MPx) activity (8).

Cannabinoids exert their therapeutic benefits in a wide range of medical conditions. Endogenous or synthetic cannabinoid receptor agonists mediate their pharmacological effects by specific cannabinoid receptors, CB1 and CB2. Both receptors are participants of the superfamily of known G-protein-coupled receptors (GPCR) (9, 10). CB1R is found in most parts of the GI tract, with the greatest concentrations in the colon and stomach (11). CB2R expression is found in immune cells, as well as the enteric nervous system (ENS) (12).

 Cannabinoids decrease stomach acid production and hence have a powerful influence on the GI tract (13), inhibiting emesis and decreasing lower esophageal sphincter pressure (14, 15). Recent studies in the gut have found an interaction between cholecystokinin and cannabinoids in the control of feeding behavior and obesity (16). Moreover, clinical studies showed that inhibition of the ‘endocannabinoid system’ (ECS) in the gut was a part of the pathogenesis of inflammatory bowel disease (17). Furthermore, CB1 receptor activation revealed immunohistochemistry location of CB1 receptors on the neurons containing acetylcholine that innervate mucosa and the blood vessels of submucosa in rat stomach (18).

This study was carried out to explain the mechanism(s) of the gastric protective effects of cannabinoid agonists and antagonists in the WIRS-induced ulcer model. The possible modulatory effects of agonists and antagonists for CB1 and CB2 receptors was assessed on some aggressive as well as protective defense factors in stomach mucosa of rats taking cannabinoids.

## Materials and Methods

***Animals***

Adult male Sprague-Dawley rats weighing 140–150 g were from the National Research Centre (NRC) Animal House Colony, Egypt; they were placed under regular housing circumstances (60% humidity and 24-27 °C room temperature with alternating 12 hr cycles of light and dark). Water and standard food were provided *ad libitum*. All procedures were carried out in accordance with the protocol approved by the NRC (Giza, Egypt) which was obtained before starting the experiment (certificate no. 14145) and according to the Ethics Committee, Faculty of Pharmacy, Cairo University (no PT 1262). 

***Drugs and chemicals***

CB1-receptor agonist NADA (N-arachidonoyl dopamine), CB1-receptor antagonist Rimonabant (SR141716A), CB2-receptor agonist GW 405833( 1,2,3-Dichlorobenzoyl)-5-methoxy-2-methyl-3-[2-(4-morpholinyl)ethyl]-1H-indole), and CB2-receptor antagonist AM630 (6-Iodopravadoline) were all from Sigma-Aldrich Chemical, USA. Dimethyl sulfoxide (DMSO) was used to dissolve the agonists and antagonists. 

***Experimental design***

Animals were located in six groups (6 rats each) as follows: 

**Group 1** :(vehicle): rats were given DMSO (0.1 ml, SC).

**Group 2**: (WIRS): rats were given DMSO (0.1 ml, SC) 30 min before the WIRS-induced ulcer. 

**Group 3-6**: All agonists and antagonists of the cannabinoid receptors were given in the dose of (1 mg/kg SC in DMSO) 30 min before WIRS-induced ulcers.

**Methods**

***Gastric mucosal injury studies***

*WIRS-induced gastric ulcer model*

The water-immersion-restrain-stress-induced ulcer model requires animals to fast for 24–36 hr but have free admission to tap water before the beginning of the experiment. After that, ulcers are produced by confining the animals in a restricted cage (17 cm (H) ×4 cm (W)× 7 cm (L)), and immersing them vertically in a water tank (15–20 °C) for 4 hr to the level of xiphoid to prevent drowning, after completion of the stress, animals were then sacrificed and all stomachs were immediately removed. The stomachs were opened in the direction of the great curvature, cleaned, stretched out on a flat surface, and fixed with 10% formalin (19).

***Assessment of gross mucosal damage***

*Determination of ulcer number and severity*

The stomach was opened and fastened to a piece of plastic board along the greater curvature. Mucosal necrotic lesions and red erosions were examined on the mucosa. (20). The total number of ulcers was counted, and then the severity of the ulcers was assessed using the following scores:

0= no ulcer, 1 = lesion size ≤ than 1 mm, 2 = lesion of size 1-2 mm, 3 = lesion of size 2-3 mm, 4 = lesion of size 3-4 mm and 5= lesion of size > 4 mm.

*Biochemical assessment*

The stomach was immediately put over the ice-cold surface after gross lesion evaluation. The glandular mucosa was cut and washed by an ice-cold saline solution (0.9 % NaCl), weighed, and stored at (-80 °C). moreover, the tissues were homogenized using a Glas-Col homogenizer (Terre Haute, USA) with 0.1 M phosphate buffer saline (PBS) at pH 7.4 to obtain 20% w/v for the biochemical tests.

***Determination of lipid peroxidation***

The level of MDA was used to measure lipid peroxidation in gastric mucosal homogenates. Determination of MDA was by measuring thiobarbituric reactive species using the Uchiyama and Mihara method in which the thiobarbituric acid reactive substances react with thiobarbituric acid to give a red-colored complex having peak absorbance at 532 nm. The amount of lipid peroxidation was measured in nmol of MDA per gram tissue (21).

***Determination of GSH***

Ellman’s method was used to determine GSH. The procedure is based on Ellman ´s reagent reduction by–SH groups of GSH to form 2-nitro-s-mercaptobenzoic acid, the nitromercaptobenzoic acid anion which has a bright yellow color that can be detected spectrophotometrically at 412 nm. The amount of GSH in the stomach homogenate was measured in µmol per gram of tissue (22). 

***Determination of PON-1 activity***

 The arylesterase activity of PON-1 was measured spectrophotometrically by using phenylacetate. The cleavage of phenylacetate was catalyzed by PON-1 to produce phenol, which can be quantified using a Recording Spectrophotometer by measuring the increase in absorbance at 270 nm and 25 °C (Shimadzu Corporation). One unit of arylesterase activity is referred to as 1 μmol/l of phenol formed per minute. The activity of enzymes was estimated using the extinction coefficient of phenol of 1310 mol/cm at 270 nm, 25 °C, and pH 8.0 and then expressed as (kU/l) (23).

***Determination of NO***

Griess reagent was used to quantify NO as nitrite in tissue homogenates. Nitrate is converted to nitrite via nitrate reductase. The Griess reagent then converts nitrite to an azo compound that can be measured using a spectrophotometer (24). At 540 nm, the chromophoricazo derivative may be colorimetrically detected. The amount of total nitrite/nitrate (NOx) in each gram of tissue was measured in micromoles.

***Pro-inflammatory cytokine assessment***

*Determination of tumor necrosis factor-alpha*

TNF-α was measured in gastric homogenates using a commercially available enzyme-linked immunosorbent assay (ELISA) kit (K0331196, Koma biotech, Gangseo-gu Seoul, Korea). All standards and samples were pipetted into wells containing immobilized antibodies specific for rat TNF-α and incubated at 37 °C for 30 min. The chromogens A & B were added to the wells and incubated at 37 °C for 15 min; the development of color is proportional to the TNF-α amount bounded. Color intensity was measured at 450 nm after 10 min.

*Determination of Interleukin-1β*

An enzyme-linked immunosorbent assay (ELISA) commercial kit (CEK1772, Cohesion Biosciences, London, UK) was used to measure IL-1 in stomach homogenate. 100 l of each standard and sample should be placed in the corresponding wells, covered tightly and incubated at room temperature for 90 min at 4 °C with moderate shaking. After removing the lid and discarding the solution, the plate was washed three times with the wash buffer working solution, allowing the solution to sit in the wells for 1 to 2 min each time. Using paper towels or another absorbent material blot the plate. At no point should the wells be entirely dry. In each well, pour 100 μl of biotin-labeled detection antibody working solution and incubate for 60 min at 37 °C. Wash the plate three times with a wash buffer working solution, allowing the solution to sit in the wells for one to two minutes each time. Pour off the wash buffer solution and wipe the plate with paper towels. In each well, pour 100 μl of Streptavidin-HRP working solution and incubate for 45 min at 37 °C. Wash the plate five times with the wash buffer working solution, allowing the wash solution to sit in the wells for 1 to 2 min each time. Remove the wash buffer from the plate and wipe it dry with paper towels or other absorbent material. In each well, pour 100 μl of TMB substrate solution and incubate it for 30 min at 37 °C in a dark place. Fill each well with 100 μl of stop solution. The color turns yellow almost instantly; the color intensity was measured at 450 nm after 10 min.

***Neutrophil infiltration assessment***

*Determination of myeloperoxidase*

Estimation of MPO in stomach homogenate was done by using enzyme-linked immunosorbent assay (ELISA) commercial kit (HK105-02, Hycult ®biotech, Uden, Netherlands). Before use, all reagents and samples were warmed to room temperature (20–25 °C). In the relevant wells, 100 μl of each standard and sample were introduced. The plate was firmly covered and incubated for 1 hr at room temperature. After discarding the solution, the wells were cleaned four times with a wash solution. Washing was accomplished by using a multi-channel pipette to fill each well with wash buffer (200 μl). Achieving good performance requires complete liquid elimination at each phase. Any leftover wash buffer was removed by flipping the plate against clean paper towels after the last wash. Each well received 100 l of diluted tracer. The plate was incubated for 1 hr with moderate shaking. The wash was repeated after the solution was dumped. Each well received 100 l of prepared Streptavidin solutions. At room temperature, the plate was incubated for 1 hr with moderate shaking. The solution was removed after the incubation period and the wash was repeated. After that, each well-received 100 l of TMB one-step substrate reagent. At room temperature, the plate was incubated also for 20–30 min. Each well received 100 l of stop solution. After adding a stop solution at 450 nm, the optical density was measured within 30 min.

*Examination of histopathology*

The stomachs of separate groups were dissected and preserved in (10%) formalin. Because formalin has a good hardening effect and produces less tissue shrinking, it was chosen. Dehydration in escalating degrees of alcohol (70 percent, 90 percent, and three changes in absolute alcohol) was followed by fixation for one or two days, clearing with xylene, and to create solid blocks containing the tissue, three consecutive changes in soft paraffin at 50 °C were used, followed by embedding in paraffin wax. Seven m thick serial transverse sections were cut. Paraffin slices were attached to albumin glycerin-coated slides and stained with hematoxylin and eosin. Light microscopy was used to assess the quality of hematoxylin and eosin sections (25).

*Determination of inflammatory neutrophils count*

A computer system using the Leica Qwin Plus software package version 3 (Switzerland) was used to count neutrophils in 5 fields in each group. Light microscopy at a magnification of 400 was used to transmit the image to the monitor screen.

***Statistical analysis***

The mean and standard error (SE) of the data were calculated. GraphPad Prism 5 Software version 5 (SanDiego, CA, USA) was used for statistical analysis. Using one-way analysis of variance test (ANOVA) followed by Dunnett’s multiple comparisons test. Ulcer scores were performed by Kruskal-Wallis non-parametric one-way analysis of variance (ANOVA) followed by Dunn’s multiple comparisons test. Data were expressed as median ± Interquartile Range (IQR). The significance criteria were set at 0.05 level of probability. Excel was used to create graphical representations and perform regression analysis.

**Figure 1:**
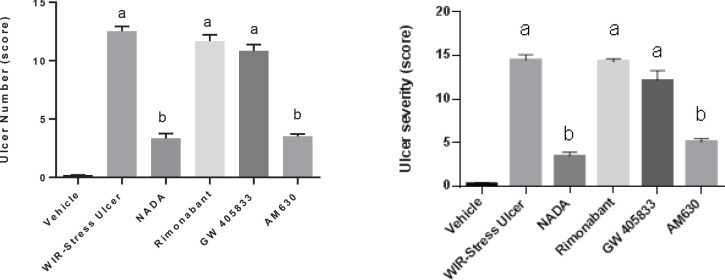
Effect of cannabinoids on gastric mucosal number and severity in rats after gastric ulcers induced by WIR-stress

Data were expressed as median ± IQR. (n=6). Statistical analysis was carried out by Kruskal-Wallis non-parametric one-way analysis of variance (ANOVA) followed by Dunn's multiple comparisons test. A) Significantly different from the vehicle group at *P*<0.05. b) Significantly different from WIR- Stress ulcer group at *P*<0.05

**Table 1:** Effect of cannabinoid agonists and antagonists on gastric mucosal biomarkers of oxidative stress after induction of gastric ulcer by WIR-stress in rats

**PON-1** **(mU/g tissue)**	**NO** **(μmol/g tissue)**	**MDA** **(nmol/g tissue)**	**GSH** **(μmol/g tissue)**	** Parameter** **Group**

23.8±2	11.28±1.6	253.1±27	2.58±0.22	**Vehicle**
7.2±0.6^a^	18.4±1.4^a^	788.5±84^a^	1.68±0.1^a^	**WIR-Stress ulcer **
19.6±1.4^ b^	8.3±.5^ b^	264.2±23^b^	2.37±0.09^b^	**WIR-Stress ulcer +NADA**
6.1±0.5^a^	16±1^a^	798.7±43^a^	1.79±.1^a^	**WIR-Stress ulcer +Rimonabant**
8.1±0.7^a^	16.6±1.2^a^	688.9±62^a^	1.84±0.14^a^	**WIR-Stress ulcer +GW 405833**
18.3±1.3^b^	10.6±0.9^b^	323±37^b^	2.35±0.021^b^	**WIR-Stress ulcer +AM630**

Data were expressed as mean ± SE (n=6). Statistical analysis was done using one-way ANOVA followed by Dunnett's test for multiple group comparisons. A) Significantly different from the vehicle group at *P*<0.05. b) Significantly different from the WIR-Stress ulcer group at *P*<0.05

GSH: Glutathione; MDA: Malondialdehyde; NO: Nitric oxide; PON-1: Paraoxonase-1

**Figure 2:**
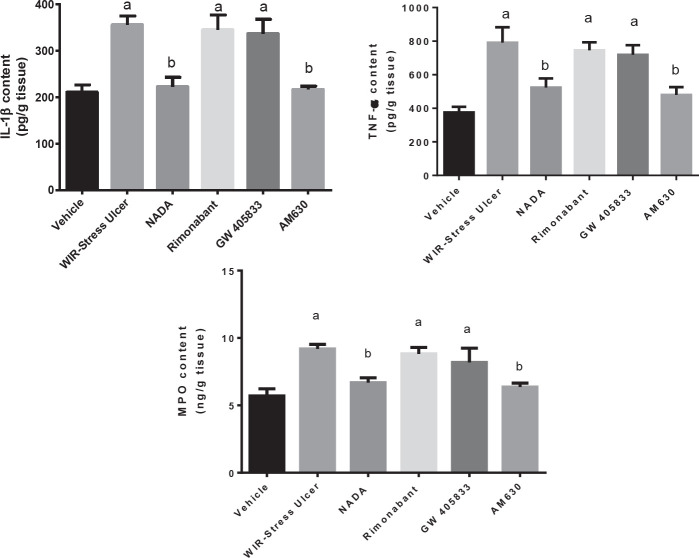
Effect of cannabinoids in gastric mucosal pro-inflammatory cytokine and Neutrophil infiltration after WIR-stress induced gastric ulcer in rats

Data were expressed as mean ±S E (n=6). Statistical analysis was done using one-way ANOVA followed by Dunnett's test for multiple group comparisons. A) Significantly different from the vehicle group at *P*<0.05. b) Significantly different from WIR-Stress ulcer group at *P*<0.05

**Table 2:** Effect of cannabinoids on gastric mucosal inflammatory neutrophil count in rats after induction of gastric ulcer by WIRS

**Inflammatory neutrophil count**	** Parameter**
	**Group**
0	**Vehicle**
30.6±1^ a^	**WIR-stress ulcer **
4.4±0.05^ b^	**WIR-stress ulcer +NADA**
25.6±0.92^ a^	**WIR-stress ulcer +Rimonabant**
22.5±4^ a^	**WIR-stress ulcer +GW 405833**
6.2±0.48^b^	**WIR-stress ulcer +AM630**

Data were expressed as mean ± SE (n=6). Statistical analysis was done using one-way ANOVA followed by Dunnett's test for multiple group comparisons. A) Significantly different from the vehicle group at *P*<0.05. b) Significantly different from WIR-Stress ulcer group at *P*<0.05

**Figure 3:**
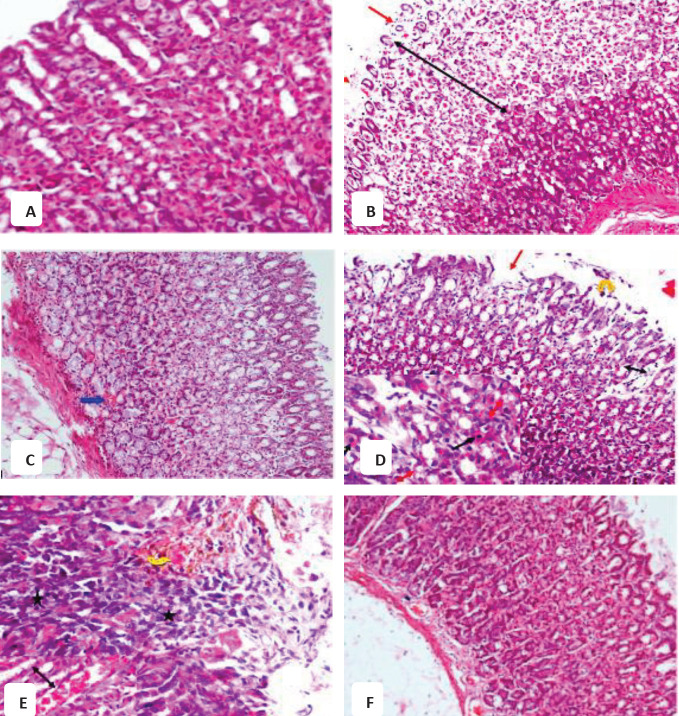
Representative photomicrographs of rat gastric mucosa sections after treatment with (A) vehicle showing normal structure; (B) WIR-stress plus vehicle showing a highly extensive gastric lesion in the form of gastric damage in the gastric mucosa, destruction, and shedding of the upper 2/3 of the gastric gland at this area; (C) WIR-stress plus NADA showing the normal architecture of the gastric gland, but the congestion of blood vessels could be observed; (D) WIR-stress plus rimonabant showing focal erosive ulcer (red arrow) and exfoliation of the focal area of superficial epithelium cells (yellow arrow) vacuolar degeneration in the epithelium lining of the gland (double red arrow). Some cells of the gastric gland appear with pyknotic nuclei while others appear with fading nuclei at the left of the figure; (E) WIR-stress plus GW 405833 showing inflammatory infiltrates (star) and hemorrhage (yellow arrow) and congested blood vessels in lamina propria (black arrow); (F) WIR-stress plus AM630 showing some improvement in histological changes in the form of no ulcer observed (H&E x200)

**Figure 4:**
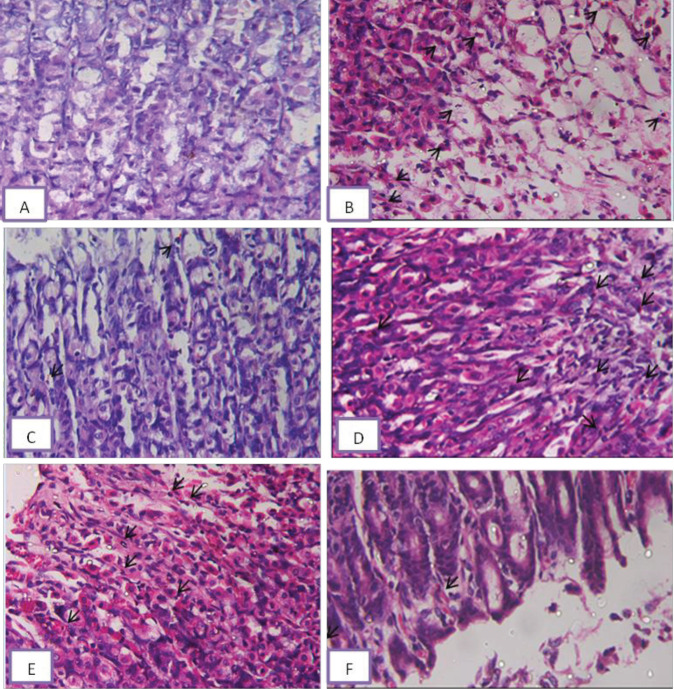
Representative photomicrographs of section of rat gastric mucosa after treatment with (A) vehicle no neutrophils seen in these lesions; (B) WIR-stress plus vehicle ulcerative gastric mucosa of rats under the effect of WIR-stress ulcer showed mucosal congestion and severe neutrophils (arrows) had infiltrated into these lesions; (C) WIR-stress plus NADA showed mild neutrophils (arrows) infiltration; (D) WIR-stress plus rimonabant showed sever neutrophils (arrows) infiltration into these lesions; (E) WIR-stress plus GW showed moderate neutrophils (arrows) infiltration into these lesions; (F) WIR-stress plus AM630 showed mild neutrophils (arrows) infiltration into these lesions (H&E x400)

## Results

***Effect of cannabinoids on gastric lesions in rats after induction of gastric ulcers by WIR-stress***

*Ulcer number and severity*

In this study, water immersion-restraint stress resulted in a gross inspection of the glandular segment of the stomach of the WIRS treated group that produced gastric mucosal damage shown as elevated ulcers number and ulcers severity. Treatment with NADA (CB1agonist) and AM630 (CB2 antagonist) reduced this number by 74% and 71.2%, respectively, as compared with the WIRS group. Moreover, Treatment with NADA (CB1agonist) and AM630 (CB2 antagonist) reduced ulcers severity by 75% and 65.5%, respectively, as compared with the WIRS control group (Figure 1).

***Effect of cannabinoids on gastric mucosal oxidative stress***

Exposure to WIRS caused a significant elevation in the content of gastric mucosal MDA and NO contents by 211.6 % and 63.1%, respectively, along with a significant decrease in GSH content and PON-1 activity by 34.8 % and 69.6 %, respectively, when compared with the vehicle group. Rats subjected to the CB1 agonist NADA and the CB2 antagonist AM630 decreased lipid peroxidation and enhanced the specific mechanisms of anti-oxidants in rats. MDA content in the mucosa decreased significantly by 66.4% and 59%, respectively, GSH level significantly increased by 40.5% and 39.6%, respectively, NO content significantly decreased by 54.5%, 42.3%, respectively, while PON-1 activity showed a marked increase by 171% and 152.4%, respectively, compared with the WIRS group (Table 1).

***Effects of cannabinoids agonists and antagonists on gastric mucosal pro-inflammatory cytokines and neutrophil infiltration after induction of gastric ulcers by WIR-stress***

Induction of ulcers by WIR-stress resulted in a considerable rise in mucosal content of TNF-α and IL-1β by 110% and 68.7%, respectively, along with an elevation in mucosal MPO by 61.2% compared with the vehicle group, on the other hand, when rats were given NADA, a CB1 agonist, and AM630, a CB2 antagonist, both exhibited a significant reduction in TNF-α by 33.7% and 35.7%, respectively, with a decrease in IL-1β by 35.5% and 37.7% and MPO content decreased by 24.9% and 27.6 %, as compared with the WIRS group (Figure 2).

***Effect of cannabinoids on gastric mucosal inflammatory neutrophil count***

Induction of gastric ulcers by WIRS was associated with an elevated inflammatory neutrophil count compared with the vehicle group. Rats that took NADA (CB1 agonist) and AM630 (CB2 antagonist) had significantly reduced inflammatory neutrophil count by 85.3% and 79.7%, respectively, when compared with the WIRS group. On the other hand, rimonabant (CB1 antagonist) administration and GW 405833 (CB2 agonist) did not affect neutrophil count compared with the WIRS group (Table 2).

***Histopathological findings***

Sections of the stomach mucosa of rats treated with vehicle stained with hematoxylin and eosin showed normal structure (Figure 3A). Sections of the stomach of rats subjected to WIR-stress ulcers showed a highly extensive gastric lesion in the form of gastric damage in the gastric mucosa, destruction, and shedding of the upper top two-thirds of the gastric gland at this area (Figure 3B). The stomach mucosa of rats that had been exposed to WIR-stress ulcers with NADA showed the normal architecture of the gastric gland, but congestion of blood vessels was observed (Figure 3C). The section in the gastric mucosa having ulcer of rats under the effect of WIR-stress ulcer with rimonabant ulcer showing focal erosive ulcer and exfoliation of the focal area of superficial epithelium cells vacuolar degeneration in the lining epithelium in the gland. Some cells of the gastric gland appear with pyknotic nuclei while others appear with fading nuclei at the left of the figure (Figure 3D). Section of the ulcerated mucosa of rats under the effect of stress with Gw 405833 induced inflammatory infiltrates and hemorrhage and congested blood vessels in lamina propria is shown in Figure 3E. Section of gastric mucosa under the effect of WIR-stress ulcer with Am630 induced some improvement in histological abnormalities, as evidenced by the absence of ulcers (Figure 3F).

Sections of the gastric mucosa of rats administrated vehicle showed no neutrophils in these lesions (Figure 4A). The section in the ulcerative gastric mucosa of rats under the effect of WIR-stress ulcers showed mucosal congestion and severe neutrophils had infiltrated into these lesions (Figure 4B). The section in the ulcerative gastric mucosa of rats under the effect of WIR-stress ulcer plus NADA showed mild neutrophils infiltrated (Figure 4C). The section in the ulcerative gastric mucosa of rats under the effect of WIR-stress ulcers plus rimonabant showed severe neutrophils infiltrated into these lesions (Figure 4D). The section in the ulcerative gastric mucosa of rats under the effect of WIR-stress ulcer plus Gw 405833 showed moderate neutrophils infiltrated into these lesions (Figure 4E). The section in the ulcerative gastric mucosa of rats under the effect of WIR-stress ulcer plus AM630 showed mild neutrophils infiltrated into these lesions (Figure 4F).

## Discussion

In the present study induction of gastric ulcers after 4 hr of water immersion and restraint stress (WIRS) severe mucosal oxidative stress is shown. WIRS significantly increased the concentration of MDA and NO and reduced the activity of PON-1 and GSH content in gastric mucosa. Overproduction of ROS causes oxidative damage, which can end in cell death (26). 

Our results revealed that the gastroprotective effects of CB1R agonist NADA and CB2R antagonist AM630 involve the reduction of lipid peroxidation and enhancement of the activity of anti-oxidative enzymes by increasing GSH and the activity of PON-1, with decreasing contents of MDA and NO. In the development of stress-induced ulcers, gastric acid plays a key role (27, 28). Cannabinoids showed inhibition of gastric acid secretion so the antiulcer effect of NADA and AM630 may be linked to their antisecretory properties by CB1 receptor activation *in vivo*. Administration of NADA and AM630 decreased secretion of acid as well as the gastric damage in 2- deoxy-d- glucose-stimulated secretion of gastric acid and pepsin(29). Anandamide and other CB1 receptor agonists exhibit gastroprotective effects after peripheral and central. WIN 55,212-2 the cannabinoid receptor agonist exerts antiulcer action and this effect is likely mediated by cannabinoid CB1, but not the cannabinoid CB2 receptors, as it was prevented by using the cannabinoid CB1 receptor antagonist SR141716A, but not by the cannabinoid CB2 receptor antagonist SR144528 (30). Moreover, in line with our study, administration of anandamide reduced the ulcer area and generation of MDA in the gastric mucosa (31).

In numerous disease processes where increased oxidative stress was seen, serum PON1 activity decreased, e.g., dementia and Alzheimer’s disease (32, 33), bronchial asthma (34), and coronary heart disease. In line with our results, administration of 20 mg/kg of cannabis increased PON1 activity in thioacetamide-treated rats (35). 

CB1R activation results in a decrease in cyclic AMP cellular levels and inhibits PKA (36, 37), its ROS production regulation has been documented in a variety of systems (38) by generating enzymes such as cyclooxygenase and nNOS (39); on the other hand, endocannabinoids may also provide protection by activating CB1R, which causes the production of anti-oxidant enzymes (40). This is the means by which CB1R activation inhibits the production of ROS (40, 41).

ROS act as second messengers of different proinflammatory genes (42). pro-inflammatory cytokines increase in stomach mucosa of rats exposed to WIRS (43). In the current study, we discovered that a stressful situation was the outcome in a higher MPO content and a marked increase in the inflammatory neutrophil count compared with the normal group, while rats treated with the CB1R agonist NADA and CB2R antagonist AM630 had significantly reduced TNF- α, IL-1 β, and MPO compared with the stress ulcers group. This reduction of MPO content and neutrophils count is most likely mediated by NADA and AM630 due to a decline in the inflammatory levels of cytokines, for example, IL-1 β, IL-6, and TNF- α involved in neutrophil recruitment. THC decreased TNF- α, (44, 45), moreover, cannabidiol reduced the release of pro-inflammatory mediators (46). 

Activated neutrophils play a key role in WIRS-induced gastric mucosal ulcers (47). In the inflammatory reaction, neutrophils once activated caused the release of oxygen-derived free radicals and MPO (48, 49). MPO causes oxidative damage by producing hypochlorous acid from hydrogen peroxide and the chloride anion (50).

## Conclusion

In summary, in the current work, whereby the gastric mucosa is exposed to stress, the CB1R agonist, NADA, and CB2R antagonist, AM630 have a protective role by exerting potent anti-oxidant and anti-inflammatory effects by decreasing gastric damage, reducing mucosal oxidative stress, and decreased levels of neutrophils and cytokines. 

## Author’ Contributions

RE, RMA, OMEA and MMK Study conception or design; RE Data analyzing and draft manuscript preparation; RE, RMA and OMEA Critical revision of the paper; RMA, OMEA, MMK and ZAE Supervision of the research; RE, RMA, OMEA, MMK, NAS, ZAE and FAM Final approval of the version to be published.

## Conflicts of Interest

The authors declare that there are no conflicts of interest.
